# Study of the snail intermediate hosts of urogenital schistosomiasis in Niakhar, region of Fatick, West central Senegal

**DOI:** 10.1186/s13071-015-1030-z

**Published:** 2015-08-07

**Authors:** Bruno Senghor, Omar Talla Diaw, Souleymane Doucoure, Mouhamadane Seye, Idrissa Talla, Adiouma Diallo, Cheikh Tidiane Bâ, Cheikh Sokhna

**Affiliations:** Institut de Recherche pour le Développement, UMR 198 (URMITE), Campus International de Hann, IRD, BP 1386, CP 18524 Dakar, Sénégal; Département de Biologie Animale, laboratoire d’écologie et de Biologie évolutive, Université Cheikh Anta Diop de Dakar, BP 5005, Dakar, Senegal; Institut Sénégalais de Recherches Agricoles, ISRA, route des Hydrocarbures, Bel Air, BP 3120, Dakar, Senegal; Programme national de lutte contre les bilharzioses et les géo-helminthiases, ministère de la santé et de l’action sociale, Dakar, Sénégal

**Keywords:** *B. senegalensis*, *B. umbilicatus*, *S. haematobium*, Urogenital schistosomiasis, Ponds, Niakhar, Senegal

## Abstract

**Background:**

*Schistosoma haematobium* is the most widespread schistosome species in Senegal and occurs in several regions of the country especially in the Sudan-Sahelian zone. The aims of the study were i) to determine the freshwater species ii) to ascertain the role of the identified snail species in the transmission of *S. haematobium* and iii) to study the impact of drought on the snails.

**Methods:**

Snails were sampled each year in 17 sites from July to November-December 2012 and 2013. At each snail survey, snails were grouped by village, counted, identified according to shell morphology and the rates of schistosome cercarial shedding recorded. The shell height of the snails collected in July was measured and classified into four groups according to their size in order to determine those that are open to aestivation.

**Results:**

*B. senegalensis* and *B. umbilicatus* were the only snails intermediate hosts collected in the Niakhar study area. *B. senegalensis* is found in all the 17 sampling sites while *B. umbilicatus* was only found in one site out of the many surveyed. The total number of *B. senegalensis* collected in 2012 and 2013 was 1032 and 8261 respectively. A total of 901 and 6432 *B. senegalensis* were tested for *Schistosoma* spp. infestation in 2012 and 2013 respectively. For *B. umbilicatus*, 58 snails were collected and tested in 2012. In 2013, 290 were collected and 281 tested. The overall rates of schistosome cercarial shedding were 0 % in 2012 and 0.12 % in 2013 for *B. senegalensis* and 13.79 and 4.98 % in 2012 and 2013 respectively for *B. umbilicatus*. For both species collected in July, size group 3 individuals (7–9.9 mm) were the most numerous, 63.6 and 57.8 % for *B. senegalensis* and *B. umbilicatus* respectively. *B. umbilicatus* was reported for the first time in the region of Fatick located in the old ecological zone of Sine-Saloum, is able to maintain *Schistosoma* spp. larvae during 7 months of drought and may transmit the disease in early July, increasing the period and the risk of transmission.

**Conclusion:**

This study recommends an adaptation of snail control strategies at pond cycles and ecology of the snails in these seasonal foci. Malacological control strategies must take into account these phenomena of drought resistance and the capacity of some snails to maintain parasite during aestivation. The treatment of ponds with Bayluscide at the end of the rainy season in November and upon onset of rains in July would be more advantageous to the control of snails thereby reducing transmission of urogenital schistosomiasis in the Niakhar area.

## Background

Schistosomiasis is one of the most prevalent parasitic infections in tropical and subtropical regions of the world and has significant economic and public health consequences in tropical and subtropical regions [1, 2]. In 2003, 112 million people in Sub Saharan Africa were estimated infected with *Schistosoma haematobium*, the parasite responsible for urogenital schistosomiasis [3]. Freshwater gastropods of the genus Bulinus are the intermediate snail hosts of *S. haematobium* [4]. Infection by *S. haematobium* occurs during routine agricultural, domestic, occupational and recreational water-related activities where human individuals are exposed to the snails carrying the parasites.

Previous malacological studies on temporary rain-ecosystems in West Africa [5–7] and in central Africa [8] have shown the importance of ponds, representing the habitats of the snail, the intermediate host of *S. haematobium.* The parasite *S. haematobium* is the most widespread schistosome species in Senegal and occurs in all 13 regions of the country except Dakar. *Bulinus senegalensis, Bulinus globosus, Bulinus truncatus* and *Bulinus umbilicatus* are the species involved in the transmission of *S. haematobium* [9, 10], but their seasonal dynamics and the role of each species varies from one ecological area to the next. In the most part of the country, transmission sites consist of temporary ponds and their occurrence depends on rainfall. In the region of Tambacounda, malacological studies showed that *B. senegalensis* and *B. umbilicatus* are the most common species and these two snails can survive for 6 to 8 months when ponds are dry [7]. In other localities of the regions of Fatick and Kaolack, represented the ecological zone of Sine-Saloum situated in the Soudan-Sahelian domain, only the presence of *B. senegalensis*, *Bulinus forskalii B. globosus* and *B. truncatus* have been reported [11].

The Niakhar area is located in the Fatick region and is endemic for urogenital schistosomiasis with prevalence of 57.6 % [12]. No information is available about the intermediate hosts of *S. haematobium* in this area and it was for this reason that we conducted this study. The aims of the study were i) to determine the freshwater species ii) to ascertain the role of the identified snail species in the transmission of *S. haematobium* and iii) to study the impact of drought on the snails.

## Methods

### Study area

**Fig. 1:**
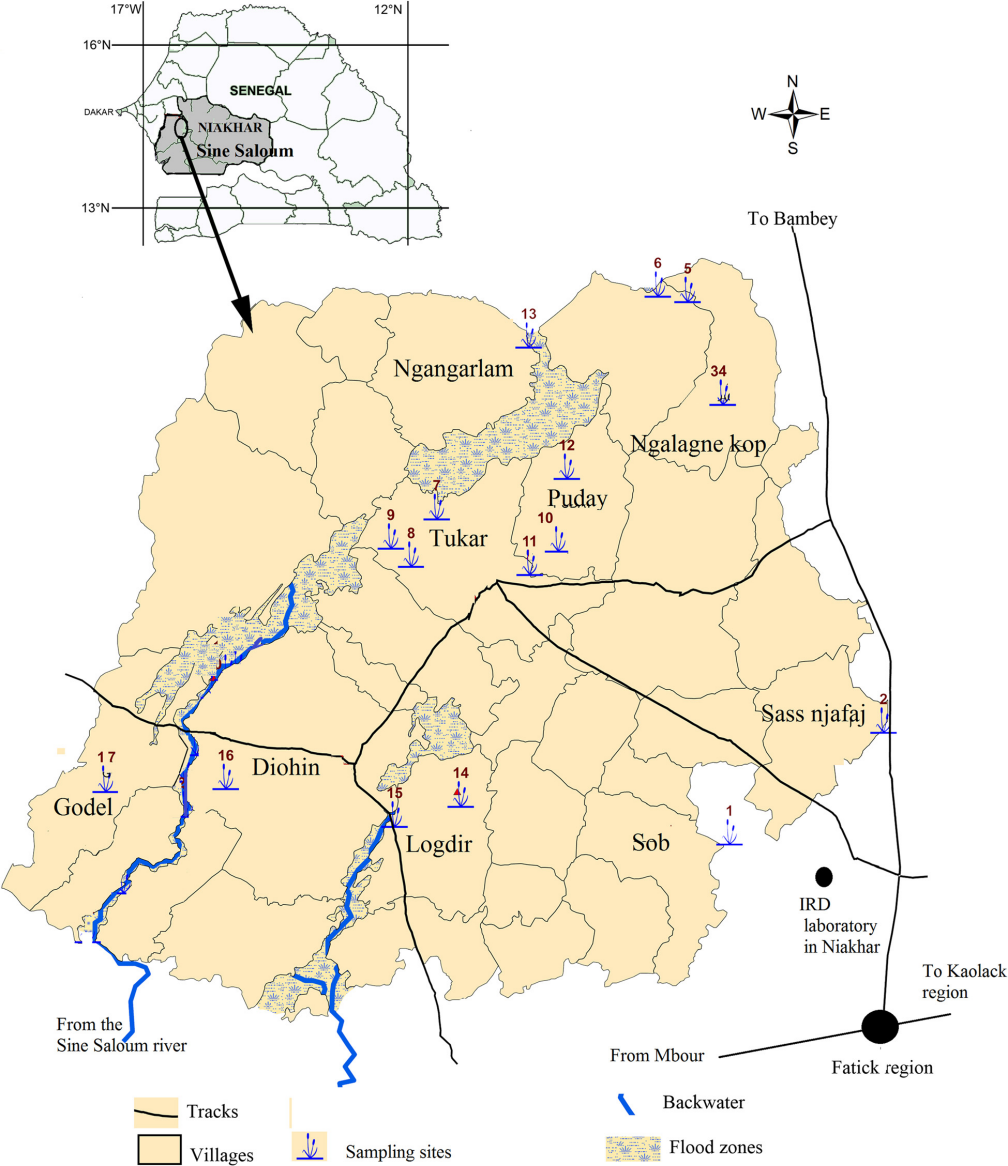
Map of the study area of Niakhar showing snail sampling sites

**Fig. 2:**
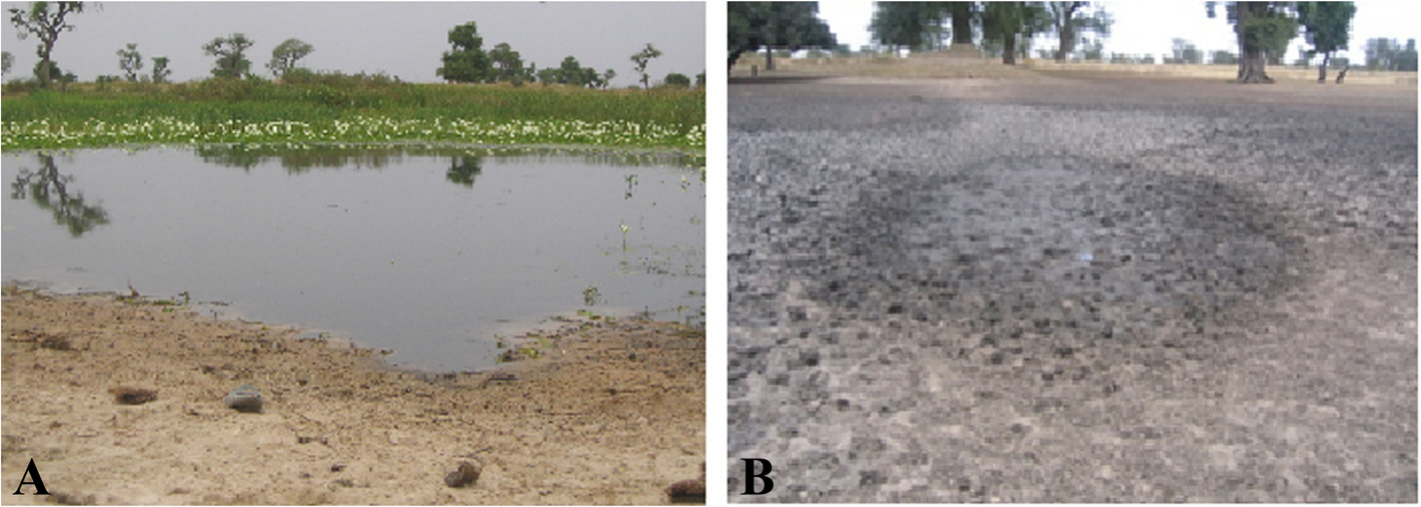
Pictures showing the water bodies of the Niakhar area: a filled pond from July to November (**a**) and a dry pond from December to June (**b**)

The study was carried out in Niakhar (14°30 N, 16°30 W), a rural area located in the Fatick region (Sine-Saloum), in central Senegal, 135 km east of Dakar, the capital of Senegal, in West Africa (Fig. 1). Niakhar is situated in a Sahelian-Sudanese climatic domain, with temperatures ranging from 24 °C in December to 30 °C in June [13]. This region’s climate is characterized by two distinct seasons: a dry season that lasts 7 to 8 months (November to May-June), and a rainy season for a period of 4 to 5 months (June-July through October). The average annual rainfall decreased from 800 mm in the 1950s to 500 mm in the 1980s. Increasing precipitation has been observed since the mid-2000s, with an average annual rainfall of 600 mm between 2005 and 2010 [14]. The area is a Health and Demographic Surveillance System of the Institute of Research for Development (IRD) in Senegal. Daily rainfall in the study area is recorded regularly for the institute by trained personnel. From 2011 to 2013, the average precipitation was 611.4 mm (Niakhar rainfall data). Many temporary ponds form during the rainy season from July to November-December and are dry from November-December to June (Fig. 2). These water bodies are used for laundry, bathing, swimming, fetching water and watering domestic animals. Nine villages: Godel, Diohin, Logdir, Ngalagne kop, Ngangarlam, Puday, Sob, Sass Njafaj and Tukar (Fig. 1) were selected for this study according to their prevalence of *S. haematobium* determined in our previous study [12, 15].

### Study sample selection and snail sampling

A preliminary investigation in these villages was conducted to identify the different ponds where people were involved in domestic and recreational activities. The geographical positions of the sites surveyed were determined using a global positioning system (GPS). A total of 17 ponds distributed in these nine villages (Fig. 1) were selected taking into account frequency of visitation by the villagers and proximity to the households.

At each selected pond, snails were sampled from July to December 2012 and 2013, when the water was present in the ponds. They were collected using a scoop net during 15 min at monthly intervals in 2012 and at 15-day intervals for 30 min in 2013 in order to have more data for better understanding the role of the snails. Collected snails from each sampling site were placed in 50 mL plastic containers and transferred within 4 h to the laboratory.

### Snail abundance, identification and determination of infestation rates

The sampled snails were rinsed with tap water, sorted and classified according to shell morphology using the field identification keys of Kristensen [16]. Snail abundance was determined by calculating the total number of snails collected per hour and per person [7]. For this, the number of snails collected in each sampling site during 15 and 30 min was multiplied by 4 and 2, respectively. Each snail was once tested by placing in glass tube with 10 mL of filtered water and exposing to direct sunlight or to electric light for 30 to 40 min to induce cercarial shedding. The schistosome cercariae issued by infested snails were then checked under a dissecting microscope and identified according to the criteria developed by Fransden & Christensen [17]. The ratio of the snail number shedding *Schistosoma* spp cercariae and the total number of snails tested represent the infestation rate.

### Snail resistance to drought

Since the ponds of the Niakhar area are temporary, holding rainwater for five to six months and dry up for a period of six to seven months, some snails disappear with the water into the mud during this drying period and reappear in July, when the first rains are sufficient to re-colonize the biotope. The shell height of the snails collected in July was measured under a dissecting microscope using a millimeter paper in order to determine those that had aestivated withstood the drought. The snails were then classified into four groups according to their size: group 1 (3–4.9 mm), group 2 (5–6.9 mm), group 3 (7–9.9 mm) and group 4 (10–15 mm). Group 1 represented young snails and groups 2, 3 and 4 the adults.

### Ethical approval

The study was a part of a larger investigation of schistosomiasis epidemiology, transmission and control in Senegal and was approved by the Senegalese National Ethics Committee (reference n° SN11/57).

## Results

### Rainfall pattern in the Niakhar area

**Fig. 3:**
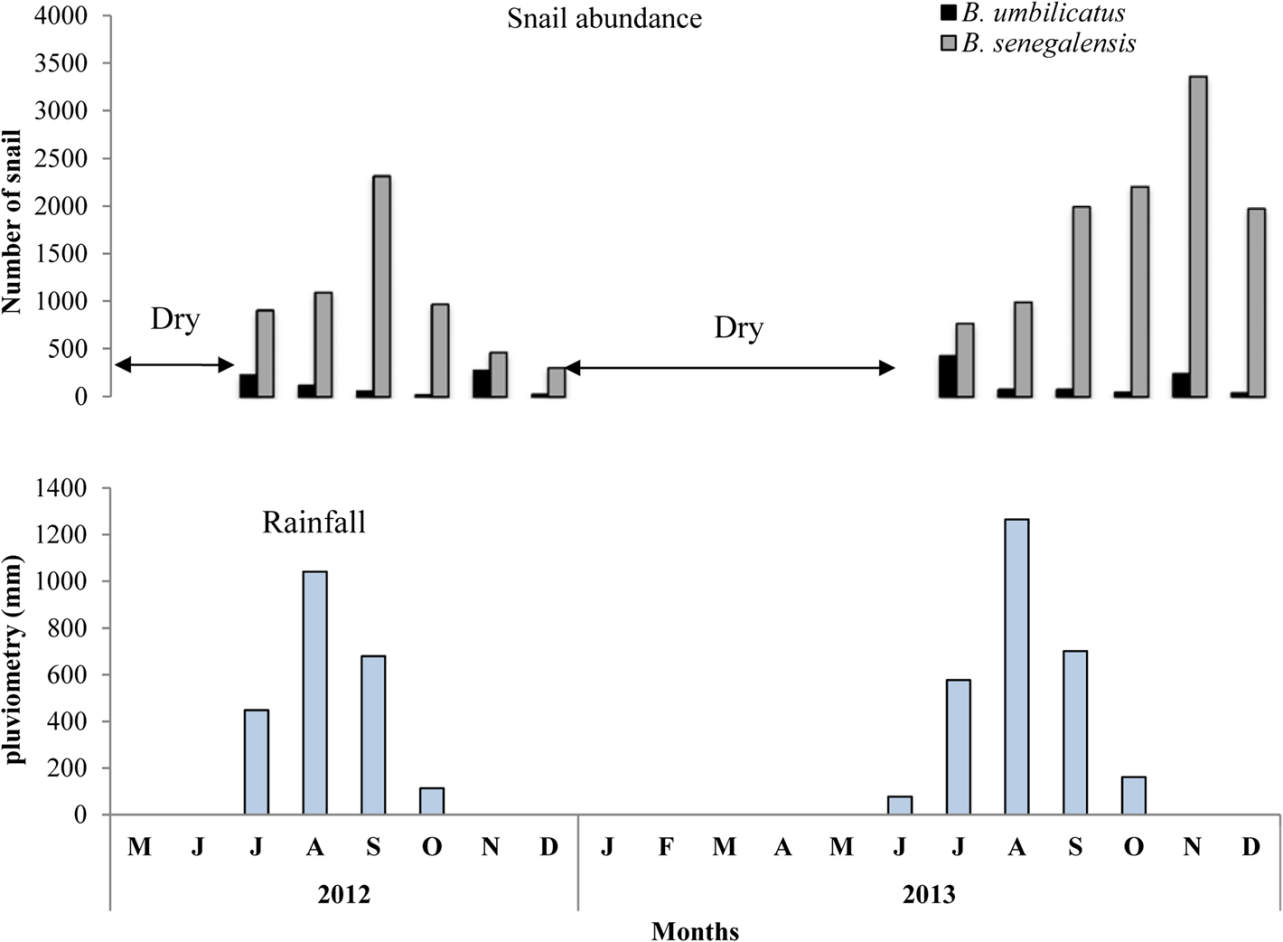
Abundance of snails in relation to rainfall in the Niakhar temporary ponds from 2012 to 2013

In 2012, the first rains occurred in July and lasted in November. The average annual rainfall was 570.3 mm. In 2013, the rains started in June and lasted in November with an average annual rainfall of 695.7 mm. During the study period in 2012 and 2013, rains were more abundant between August and September and maximum rainfall was recorded in August (Fig. 3).

### Freshwater snails in the study area, their infestation status and their dynamic

All the ponds surveyed in the nine villages during the two years of the study yielded snails during sampling. A total of 1090 and 8551 snails were collected in 2012 and 2013, respectively. Based on shell morphology, 1032 (94.7 %) and 58 (5.3 %) of the snails were identified in 2012 as *B. senegalensis* and *B. umbilicatus*, respectively. In 2013, 8261 (96.6 %) and 290 (3.4 %) *B. senegalensis* and *B. umbilicatus* were collected respectively. *B. senegalensis* is the most abundant and was always encountered in all the ponds of the nine villages, while *B. umbilicatus* was found in one pond located in the village of Ngangarlam.

The number of snails tested in 2012 and 2013 was 58 and 281 for *B. umbilicatus*, and 901 and 6432 for *B. senegalensis. B. umbilicatus* was found to be infested by *Schistosoma* spp. in the pond of the village of Ngangarlam in 2012 and 2013 with rates of infestation of 13.79 and 4.9 %, respectively. In 2012, no *B. senegalensis* infested by *Schistosoma* spp. was found. In 2013, infested *B. senegalensis* were collected from four ponds located in the villages of Sass Ndiafaj, Tukar, Puday and Logdir with rates of 0.61, 0.07, 0.05 and 1.11 %, respectively. The total rate of infested *B. senegalensis* was 0.12 % in 2013. Overall, regardless of species, infestation rates were 0.01 and 0.003 % in 2012 and 2013 respectively (Table 1).

**Table 1:** Total number of snails collected and *Schistosoma* spp. infestation rates (%) in ponds in 2012 and 2013, in 9 villages of the Niakhar study area

Villages (pond)	Snail species	2012			2013			Total		
		Col.	Test.	Inf. (rate)	Col.	Test.	Inf. (rate)	Col.	Test.	Inf. (rate)
Sob (1)	*B. s*	44	44	0	512	330	0	556	374	0
Sass Ndiafaj (1)	*B. s*	20	20	0	898	819	5 (0.61 %)	918	839	5 (0.60 %)
Ngalagne kop (4)	*B. s*	156	156	0	882	714	0	1038	870	0
Tukar (3)	*B. s*	150	150	0	1512	1337	1 (0.07 %)	1662	1487	1 (0.06 %)
Puday (3)	*B. s*	122	111	0	2912	1920	1 (0.05 %)	3034	2031	1 (0.04 %)
Logdir (2)	*B. s*	80	80	0	90	90	1 (1.11 %)	170	170	1 (0.60 %)
Diohin (1)	*B. s*	114	110	0	902	689	0	1016	799	0
Godel (1)	*B. s*	64	64	0	278	270	0	342	334	0
Ngangarlam (1)	*B. s*	166	166	0	275	263	0	441	429	0
	*B. u*	58	58	8 (13.79 %)	290	281	14 (4.98 %)	348	339	22 (6.51 %
Total	*B. s*	1032	901	0	8261	6432	8 (0.12 %)	9293	7333	8 (0.11 %)
	*B. u*	58	58	8 (13.8 %)	290	281	14 (4.98 %)	348	339	22 (6.50 %)
Total all species		1090	959	8 (0.01 %)	8551	6713	22 (0.003 %)	9641	7672	30 (0.39 %)

*B. s: B. senegalensis* - *B. u: B. umbilicatus*, *Col* Collected, *Test* Tested, *Inf* Infested

When analyzing the snails dynamic, the results show that in 2012, there was one peak for both species situated in September and November for *B. senegalensis* and *B. umbilicatus* respectively; while in 2013 two peaks were observed for *B. senegalensis* in September and November, with still one peak for *B. umbilicatus* in November (Fig. 3).

### Assessment of the impact of drought on Bulinus species and their infestation with Schistosome cercariae

In the two years, the snail populations studied in July consisted of specimens whose shell height was greater than 4.9 mm. In 2012, 69.4 % *B. senegalensis* and 55.2 % *B. umbilicatus* collected are in the size group 3. The same trends are obtained in 2013 with 60.8 % *B. senegalensis* and 66.7 % *B. umbilicatus* classified in group 3. Overall, for both species, size group 3 individuals were the most numerous, 63.6 and 62.6 % for *B. senegalensis* and *B. umbilicatus* respectively (Table 2). All the snails collected in July 2012 and 2013 were tested for cercaria shedding. Only *B. umbilicatus* from the pond of the village of Ngangarlam was infested with *Schistosoma* spp. cercariae. The infestation rates were 13.8 and 11.1 % in 2012 and 2013 respectively (Table 3).

**Table 2:** Estimation of the age of *B. senegalensis* and *B. umbilicatus* collected in July 2012 and July 2013 measured and grouped by shell size (mm)

	Shell size groups (mm)	Group 1	Group 2	Group 3	Group 4	
		(3–4.9)	(5–6.9)	(7–9.9)	(10–15)	
Snail species	Total snails collected	No. Snails (%)	No. Snails (%)	No. Snails (%)	No. Snails (%)	Years
*B. senegalensis*	203	0 (0)	37 (18.2)	141 (69.4)	25 (12.3)	2012
*B. umbilicatus*	58	0 (0)	21 (36.2)	32 (55.2)	5 (8.6)	
*B. senegalensis*	421	0 (0)	90 (21.4)	256 (60.8)	75 (17.8)	2013
*B. umbilicatus*	108	0 (0)	27 (25)	72 (66.7)	9 (8.3)	
*B. senegalensis*	624	0 (0)	127 (20.3)	397 (63.6)	100 (16.1)	All years
*B. umbilicatus*	166	0 (0)	48 (30)	104 (62.6)	14 (8.4)

**Table 3:** *Schistosoma* spp. infestation rates of *B. senegalensis* and *B. umbilicatus* collected after 6 to 7 months of drought in July 2012 and 2013 in Niakhar temporary ponds

Snail species	2012			2013			Total		
	Col.	Test.	Inf. (rate)	Col.	Test.	Inf. (rate)	Col.	Test.	Inf. (rate)
*B. sengalensis*	306	306	0	318	318	0	624	624	0
*B. umbilicatus*	58	58	8 (13.79 %)	108	108	12 (11.1 %)	166	166	20 (12.04 %)

## Discussion

This study is the first malacological survey conducted in the Niakhar study area. *B. umbilicatus* and *B. senegalensis* were the only snail species encountered in the 17 ponds sampled in this study. Previous malacological surveys done in the other localities of Fatick and Kaolack regions in the Sine-Saloum ecological zone reported the presence of *B. senegalensis*, *B. forskalii*, *B. globosus* and *B. truncatus* [11]. The presence of *B. umbilicatus* is reported for the first time in the Niakhar study area situated in the ecological area of Sine-Saloum. The fact that this species was not reported by previous studies conducted in other localities of the Sine-Saloum zone [11] could be due to its low geographical distribution. Indeed, in Niakhar, *B. umbilicatus* was only encountered in the village of Ngangarlam in one pond out of the 17 surveyed from 2012 to 2013.

In 2012, the rains were late and low quantities were recorded in the area compared to 2013. Thus the water in 2013 remained longer in the largest ponds at Sass njafaj, Puday and Diohin. This persistence of water in the ponds allowed the snails to undergo more breeding cycles before the ponds dried up. This could explain the differences in snail abundance observed at the end of the rains between 2012 and 2013. The fact that *B. umbilicatus* displayed a single peak at the end of the rainy season, contrary to *B. senegalensis*, suggests that this snail has a longer reproductive cycle. Indeed, the rapid draining of ponds in December prevents this species from reaching its second peak. *B. senegalensis* lay quickly when the pond fill in July-August, allowing them to reach the first peak in September whose progeny will give the second peak in November at the end of the rains. In general, maximum snail numbers were observed at the end of the rainy season. The fluctuations in snail abundance correlated to the drying up of ponds. The period and the number of population peaks varied between species and from one year to another. Similar observations were made in temporary ponds in the region of Tambacounda in Senegal. In these temporary habitats, the period of great egg-laying after re-emergence of the snails is situated in July-August and September where a great number of eggs are observed. These are times of great reproduction, especially from August to September and October. The population thus consists of snails of all ages [7]. In these types of habitats, the ecological conditions are also hostile to the development of many snail species. The duration of the rainy season and the quantities of water, are important elements governing the abundance and density of *B. umbilicatus* and *B. senegalensis*. These snails adapt to their environment by establishing a short cycle, allowing them to achieve high population density and to fulfill their role as intermediate hosts for *S. haematobium* [11].

In the current study, both species were found to be infested with schistosome cercariae. *B. senegalensis* was found to be infested by schistosome cercariae in four different ponds with rates varying from 0.05 % to 1.11 %. This snail is known to be involved in the transmission of *S. haematobium* in the middle valley around Podor and Matam [18] and also in the transmission of S. *bovis* in the Senegambia [19]. In Senegal, *B. senegalensis* is involved in the transmission of *S. haematobium* and not with *Schistosoma bovis* and *Schistosoma curassoni* [9, 20, 21]. According to these previous studies and the fact that it is the only freshwater snail in the ponds of Niakhar where it was found to be infested by *Schistosoma* spp. cercariae, it is clear that *B. senegalensis* plays a role in the transmission of *S. haematobium* in Niakhar.

*B. umbilicatus* is involved in the transmission of *S. haematobium* and *S. curasonni* in Senegal, and is well-adapted to these temporary ponds [7, 22]. In Niakhar, in the village of Ngangarlam, *B. umbilicatus* was also found to be infested by *Schistosoma* spp*.* in one pond in 2012 and 2013 with high infestation rates. Cercaria species, however, were not differentiated. Nevertheless, a high prevalence of 65.5 % of urogenital schistosomiasis was reported for the people in this particular village of Ngangarlam close to the *B. umbilicatus* site and this pond is rarely frequented by livestock (Senghor, personal communication). In addition, in other localities in the region of Tambacounda with similar ecology, *B. umbilicatus* was the only snail infested with *Schistosoma* spp*.* in a pond system in villages endemic for urogenital schistosomiasis [7]. Considering the epidemiology of urogenital schistosomiasis in this village, the fact that *S. bovis* and *S. curasonni* are not present in the Fatick region and that the water points are rarely or not frequented by livestock [23], we suspect that the cercariae produced by *B. umbilicatus* are those of *S. haematobium*. However, this result requires the use of molecular biology techniques to better characterize the cercariae produced by *B. umbilicatus* in order to better define its role in the transmission of *S. haematobium*. The same suggestion is necessary for the snail intermediate hosts in general and particularly for the snails of the genus Bulinus that can shelter in addition to *S. haematobium*, animal schistosomes, such as *S. bovis, S. curassoni* and others [17, 24].

The current study also showed that *B. umbilicatus* and *B. senegalensis* can withstand 7 to 8 months of drought and that it is the larger adult specimens that are able to persist. Similar observations were reported in the laboratory and in the field by previous studies in Senegal [7, 25]. This phenomenon of drought resistance is well known among African snails, especially the genus *Bulinus* [4, 19]*,* but little information is available concerning the ability of these snails to maintain schistosome larvae during this period.

In the present study, for the first time, *B. umbilicatus* was found to be infested in July 2012 and 2013, after a dry period of 7 months and also, 21 and 25 days after the first rains respectively. The same finding was reported for *Bulinus nasutus* in Tanzania after five months of drought and also, 21 days after the first rains [26]. This phenomenon is also of great importance in the epidemiology of urogenital schistosomiasis in the Niakhar study area and other seasonal transmission foci in Senegal because it can increase the risk and the period of transmission, positioned by previous studies between September and November [7, 11]. The fact that *B. umbilicatus* was found to be infested with *Schistosoma* spp. in Tambacounda [7] and in Niakhar from July to November with a high prevalence proves that even though this snail is well-adapted to these rain-temporary ecosystems and despite its limited geographical distribution, it plays a significant role in the epidemiology of urogenital schistosomiasis in Senegalese areas with only temporary ponds. Thus, extensive studies of this Bulinus are necessary because it is able to withstand drought and to maintain *Schistosoma* spp. larvae from one season to another. In addition, *B. umbilicatus* is involved in the transmission of animal schistosomiasis in Senegal [22].

Snail control strategies in these temporary habitats in Niakhar but also in other seasonal foci in Senegal, must take into account these phenomena. This study recommends an adaptation of snail control strategies to the cycles of ponds and snails. Two annual treatments of the pond with Bayluscide at the end of the rainy season in November before aestivation and upon onset of rains in July would be more beneficial. This would have the advantage to be focused in the puddles of residual waters or newly created waters and kill almost all snails. This would reduce snail density, but also decrease the risk of transmission by eliminating individual snails that have maintained the parasite during the drought.

## Conclusions

This study has found that *B. senegalensis* and *B. umbilicatus* are the only snail intermediate hosts of urogenital schistosomiasis found in the Niakhar study area. The prevalence of urogenital schistosomiasis in Niakhar, the epidemiology and the frequentation of water points, permit us to say that *B. senegalensis* and *B. umbilicatus* play a role in the transmission of *S. haematobium* in the Niakhar area. The short cycle of transmission in these areas with temporary ponds could have an impact on the epidemiology of urogenital schistosomiasis but could also present an opportunity for snail control in Niakhar area, which is not the case in the Senegal River Valley where transmission is permanent.
